# Intensity‐dependent ventilatory and cardiopulmonary patterns during exercise in trained individuals with cerebral palsy: An exploratory study

**DOI:** 10.1113/EP093890

**Published:** 2026-09-11

**Authors:** Se‐Hee Park, Hwan Kim, Min‐Goo Lee

**Affiliations:** ^1^ Department of Physiology Korea University School of Medicine Seoul Republic of Korea

**Keywords:** cardiopulmonary response, cerebral palsy, O_2_ pulse, ventilatory pattern, Clinical Research Information Service (CRIS): KCT0010575

## Abstract

Cerebral palsy (CP) is associated with altered exercise physiology, but it remains unclear how ventilatory and cardiopulmonary responses are expressed under high physiological demand in individuals with CP. Six athletes with CP (spastic CP: *n* = 3, all GMFCS III, all male, mean age 24 years; ataxic CP: *n* = 3, GMFCS I/I/III, two female, mean age 28 years) and three non‐disabled controls (CON: two female, mean age 28 years) performed a heart rate‐matched incremental rowing protocol. Oxygen uptake, minute ventilation (${\dot{V}}_{E}$), tidal volume (*V*_T_), respiratory frequency and O_2_ pulse were described across sitting rest and target heart rate stages of 120, 140, 160 and 180 bpm. Descriptive analyses with effect sizes were performed. Peak ${\dot{V}}_{O_{2}}$, recorded at the 180‐bpm stage, appeared lower in CP subtypes than in CON (spastic CP: 33.9, 44.8, 31.7; ataxic CP: 35.3, 32.3, 28.3; CON: 49.3, 39.8, 48.5 mL kg^−^^1^ min^−^^1^). With increasing exercise intensity, O_2_ pulse tended to increase progressively in CON and ataxic CP; in spastic CP, the increase appeared more attenuated. *V*_T_ appeared lower in CP than CON across all stages. At the 180‐bpm stage, spastic CP showed a narrow inter‐individual ${\dot{V}}_{E}$ range (71.4–88.3 L min^−^^1^) relative to CON (90.3–138.3 L min^−^^1^); ataxic CP showed wide variability (48.3–112.9 L min^−^^1^). Intensity‐dependent changes in O_2_ pulse and ventilatory patterning appeared to differ between CP subtypes and CON, suggesting these variables may reflect subtype‐specific exercise physiology in CP.

## Abstract (highlights)

- **What is the central question of this study?**
  In trained individuals with cerebral palsy (CP) classified by predominant motor presentation (spastic hypertonia versus ataxic dyscoordination), what intensity‐dependent ventilatory and cardiopulmonary response patterns emerge during heart rate‐matched incremental rowing exercise?
- **What is the main finding and its importance?**
  In trained individuals with CP, intensity‐dependent changes in O_2_ pulse and ventilatory patterning may suggest differences not only from non‐disabled controls but also between spastic and ataxic subtypes. O_2_ pulse trajectory and ventilatory patterning may serve as informative indicators reflecting subtype‐specific exercise physiological characteristics in CP.

## 1 INTRODUCTION

Cerebral palsy (CP) is characterized by abnormal muscle tone, impaired motor control and reduced cardiorespiratory fitness, often accompanied by altered exercise responses including lower peak oxygen uptake, blunted oxygen pulse (O_2_ pulse), and modified ventilatory patterns (Balemans et al., 2013; Rosenbaum et al., 2007). Although long‐term training may partially restore aerobic capacity toward non‐disabled levels, it remains unclear whether integrative cardiopulmonary efficiency and ventilatory regulation are preserved under high physiological demand (Kusano et al., 2001; Lauglo et al., 2016; Pelliccia et al., 2021; Runciman et al., 2015; Unnithan et al., 1998).

Spastic and ataxic CP represent distinct neuromuscular phenotypes with different constraints on movement efficiency and muscle mechanics (Compagnat et al., 2020; Manto, 2009; Unnithan et al., 1996). Whether these differences translate into subtype‐specific cardiopulmonary response patterns during high‐intensity exercise has not been systematically examined, in part because recruiting homogeneous cohorts of well‐trained athletes within defined CP sport classifications is inherently difficult.

O_2_ pulse reflects stroke volume and peripheral oxygen extraction according to the Fick principle (Bhambhani et al., 1994; Whipp et al., 1996), whereas ventilatory variables describe breathing strategy and mechanical efficiency during exercise (Nicolò et al., 2018). Subtype‐specific divergence in these responses may emerge at higher intensities despite similar submaximal performance, but direct observational data in trained individuals with CP remain scarce.

This study therefore aimed to characterize intensity‐dependent ventilatory response (such as minute ventilation, tidal volume [*V*_T_], respiratory frequency [RF]) and cardiopulmonary response (${\dot{V}}_{O_{2}}$, O_2_ pulse) patterns across heart rate (HR)‐matched exercise stages in trained individuals with CP. Subtype‐specific comparisons were defined by predominant neuromotor presentation – spastic hypertonia versus ataxic dyscoordination – rather than anatomical distribution. Given the constraints of recruiting this population, the present work is explicitly framed as a pilot observation intended to generate hypotheses and inform the design of future mechanistic studies.

## 2 METHODS

### 2.1 Ethical approval

This study was conducted in accordance with the standards set by the *Declaration of Helsinki*. All procedures were approved by the Institutional Review Board of Korea University College of Medicine (IRB‐2024‐0176), and all participants provided written informed consent prior to enrolment. The study was prospectively registered at the Clinical Research Information Service (CRIS: KCT0010575).

### 2.2 Participants

Nine individuals participated, including six trained athletes with CP – all actively competing in para‐athletics within World Para Athletics (WPA) classes T/F32–T34 (seated events; lower class numbers indicate greater functional impairment) with at least 2 years of structured endurance training – and three non‐disabled controls. WPA sport classification within these classes is itself based on standardised assessment of muscle tone (Ashworth Scale) and coordination (ataxia evaluation); in the present study, CP participants were characterized by Modified Ashworth Scale (MAS), Scale for the Assessment and Rating of Ataxia (SARA), Gross Motor Function Classification System (GMFCS), and Manual Ability Classification System (MACS) assessments (Table 1). Spasticity was assessed using the MAS (0–4) at shoulder flexion, extension and abduction, and elbow flexion and extension, bilaterally; scores were averaged across directions per joint and across sides. Ataxia severity was assessed in ataxic CP participants using upper‐limb items 5–7 of the SARA: finger chase, nose–finger test, and fast alternating hand movements (each item 0–4). Gross motor function was assessed with the GMFCS and manual ability with the MACS (scored bilaterally and averaged).

**TABLE 1:** General characteristics of the participants.

Participant	Age (years)	Sex	WPA sport class	Height (cm)	Weight (kg)	BMI	Skeletal muscle (%)	Body fat (%)	GMFCS	MACS	MAS (shoulder and elbow)	SARA	Peak ${\dot{V}}_{O_{2}}$ (mL kg^−^^1^ min^−^^1^)^1^
CON‐1	26	F	—	168.2	60.0	21.2	35.2	30.0	—	—	—	—	49.3
CON‐2	26	F	—	168.4	62.4	21.8	35.6	31.2	—	—	—	—	39.8
CON‐3	33	M	—	172.0	70.0	23.5	39.9	28.6	—	—	—	—	48.5
SCP‐1	26	M	F33	151.5	67.8	29.5	41.6	26.7	3	1.5	1.3	—	33.9
SCP‐2	24	M	T34	153.6	53.7	22.8	47.7	26.8	3	1.5	1.4	—	44.8
SCP‐3	24	M	F33	158.5	50.5	20.1	42.5	25.5	3	1.0	1.0	—	31.7
ACP‐1	26	F	F33	160.5	66.4	25.8	29.1	33.9	1	2.0	—	3.0	28.3
ACP‐2	29	F	F32	160.0	60.0	23.4	27.2	30.0	1	1.0	—	2.5	32.3
ACP‐3	30	M	T34	158.0	67.2	26.9	31.4	41.9	3	1.0	—	1.5	35.3

*Note*: All values are individual participant values. Skeletal muscle mass and body fat mass are expressed as a percentage of total body weight. ^1^Peak ${\dot{V}}_{O_{2}}$ (mL kg^−^^1^ min^−^^1^) was defined as the highest value recorded during the 180 bpm stage.

Abbreviations: ACP, ataxic cerebral palsy; CON, non‐disabled control; GMFCS, Gross Motor Function Classification System; MACS, Manual Ability Classification System (scored bilaterally and averaged); MAS, Modified Ashworth Scale (assessed at shoulder and elbow flexor/extensor muscles bilaterally; SARA, Scale for the Assessment and Rating of Ataxia (upper‐limb items only); scores averaged across directions per joint and across sides); SCP, spastic cerebral palsy; WPA, World Para Athletics.

### 2.3 Exercise protocol and measurements

Participants – who were trained para‐athletes whose maximum HR during regular training had been confirmed to exceed 180 bpm – performed an incremental exercise test on a wheelchair‐adapted rowing ergometer (RowErg, Concept2, Morrisville, VT, USA) with the sliding seat removed. HR‐matched exercise stages were used rather than workload‐matched stages because, at any given external workload, individuals with CP require substantially greater metabolic demand than non‐disabled peers. Studies across multiple exercise modalities – including walking (Nardon et al., 2021; Unnithan et al., 1996) and cycle and arm crank ergometry (Kusano et al., 2001; Lundberg, 1978) – consistently demonstrate mechanical efficiency approximately 50% lower in CP than in non‐disabled controls at equivalent loads, attributable to hypertonia, involuntary movements and co‐contraction, with markedly higher inter‐individual variability. Although rowing ergometer‐specific data are unavailable, this principle is expected to generalise across exercise modalities. HR matching therefore provided a more comparable level of cardiovascular demand across participants with different neuromotor presentations.

Breath‐by‐breath oxygen uptake (${\dot{V}}_{O_{2}}$) and minute ventilation (${\dot{V}}_{E}$) were measured using a portable metabolic analyser (VO_2_ Master Pro; VO2 Master Health Sensors Inc., Vernon, BC, Canada), and HR was recorded at 1‐s intervals via chest‐strap monitoring (Polar H10; Polar Electro Oy, Kempele, Finland). Stroke rhythm was standardised using a metronome, and workload was manually adjusted so that participants entered each 2‐min exercise stage within ±5 bpm of the target HR (120, 140, 160 and 180 bpm). Each stage lasted approximately 2 min; the final 1 min of each stage was used for analysis. Although participants were required to begin each 2‐min stage within ±5 bpm of the target and were instructed to maintain this range throughout the stage, HR could subsequently increase or decrease beyond the tolerance during continued exercise (Appendix Table A1). Such deviations may have reflected disability‐related limitations in rowing technique, reduced exercise endurance, or both. Data from stages in which HR later deviated by more than ±5 bpm were not excluded. Accordingly, the target heart‐rate stages should be interpreted as an intensity‐progression framework rather than as strictly maintained physiological or workload‐matched conditions. All data were smoothed using a 5‐s rolling average.

### 2.4 Derived variables and statistics

Ventilatory variables included minute ventilation (${\dot{V}}_{E}$), *V*_T_ and RF; the term RF is used here consistent with established convention in the exercise physiology literature (Nicolò et al., 2018). Peak ${\dot{V}}_{O_{2}}$ (mL kg^−^^1^ min^−^^1^) was defined as the highest ${\dot{V}}_{O_{2}}$ value recorded during the 180‐bpm stage. O_2_ pulse was calculated as ${\dot{V}}_{O_{2}}$ (mL min^−^^1^) divided by each participant's mean HR (beats min^−^^1^) recorded during that stage.

Given the descriptive purpose of this study and the sample size of *n* = 3 per group, individual participant values are presented alongside group medians and interquartile ranges (IQR) as the primary data representation. Statistical comparisons (Kruskal–Wallis and Mann–Whitney *U*‐test) and *P‐*values are reported as supplementary reference only and should not be interpreted as confirmatory evidence; the structural minimum *P‐*values for Mann–Whitney *U* at *n* = 3 per group is 0.100, meaning that statistically significant results are unattainable regardless of effect magnitude. Effect sizes are reported as rank‐biserial correlation (|*r*|), with |*r*| = 0.1, 0.3, and 0.5 interpreted as small, medium, and large, respectively (Cohen, 2013).

## 3 RESULTS

Nine participants were included: three CON, three spastic CP and three ataxic CP. Participant characteristics are summarized in Table 1; individual values for all measured variables are provided in Appendix Table A1. Peak ${\dot{V}}_{O_{2}}$ was lower in CP participants than in CON (*P* = 0.079; Table 1).

**FIGURE 1:**
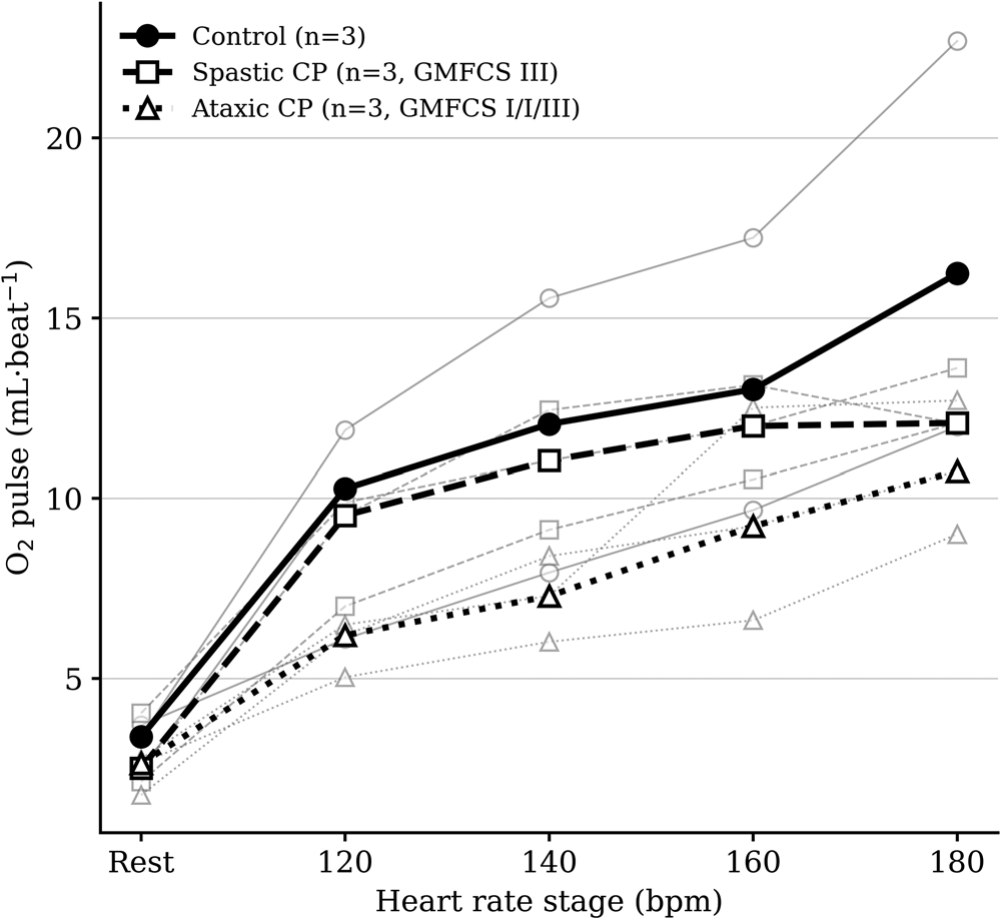
Intensity‐dependent changes in O_2_ pulse (mL beat^−^^1^) across heart rate‐matched exercise stages in non‐disabled controls (CON), spastic cerebral palsy and ataxic cerebral palsy. Bold lines with filled/open symbols represent group medians and grey lines represent individual participant trajectories.

O_2_ pulse increased progressively in CON and ataxic CP participants across all stages. Among spastic CP participants, O_2_ pulse plateaued or declined from the 160‐bpm stage onward, with one participant showing a reduction from 13.1 to 12.1 mL beat^−^^1^ between 160‐ and 180‐bpm stage (Figure 1). A Kruskal–Wallis test indicated a group difference in the magnitude of change from low to high intensity (*H* = 7.200, *P* = 0.027); pairwise comparisons did not reach the structural minimum *P‐*values at *n* = 3 per group (all *P* = 0.100), with large effect sizes for all pairs (|*r*| = 1.00). Four of nine participants deviated more than 10 bpm from the 180‐bpm target (range: 165.7–192.9 bpm); individual HR values are presented in Appendix Table A1.

**FIGURE 2:**
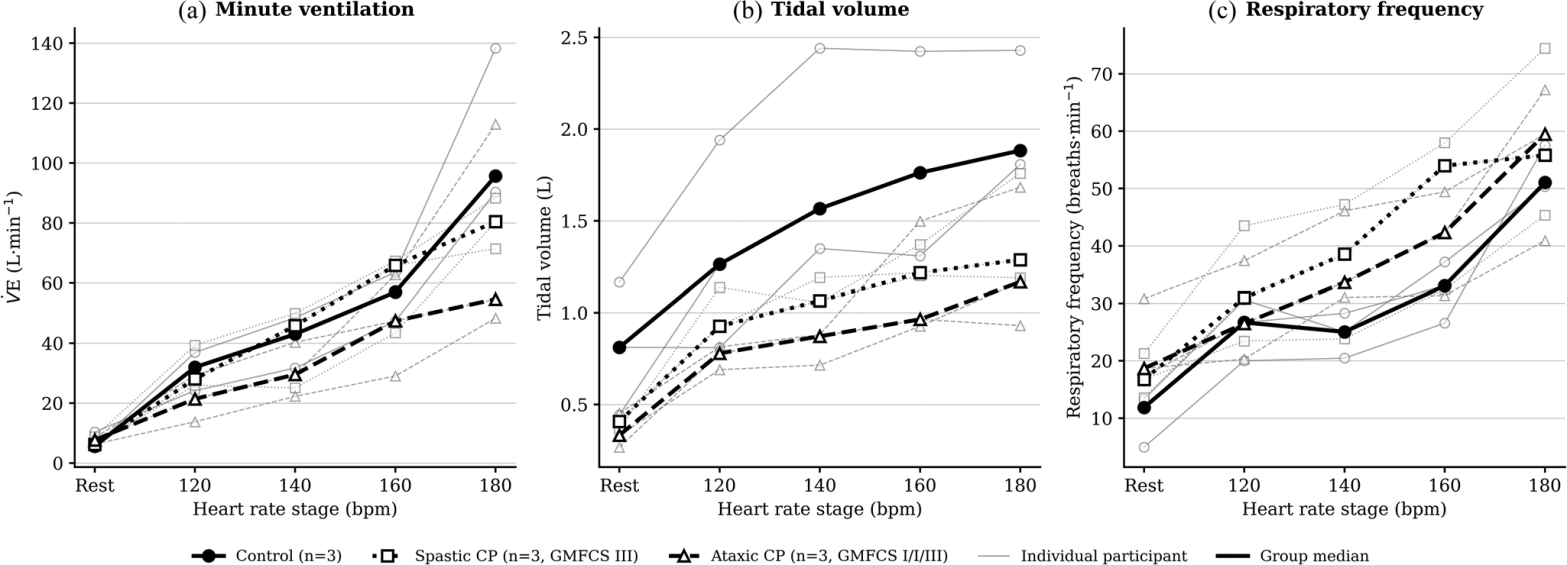
Ventilatory responses across heart rate‐matched exercise stages in non‐disabled controls (CON), spastic cerebral palsy and ataxic cerebral palsy: (a) minute ventilation (${\dot{V}}_{E}$, L min^−^^1^), (b) tidal volume (*V*_T_, L), and (c) respiratory frequency (RF, breaths min^−^^1^). Bold lines with filled/open symbols represent group medians and grey lines represent individual participant trajectories.

At rest, RF was elevated and *V*_T_ reduced in CP participants relative to CON (*P* = 0.024, |*r*| = 1.00 and *P* = 0.048, |*r*| = 0.89, respectively; Figure 2). *V*_T_ remained reduced across all exercise stages in CP participants, reaching significance at 140‐bpm stage (*P* = 0.024, |*r*| = −1.00) and 180‐bpm stage (*P* = 0.024, |*r*| = −1.00). At the 180‐bpm stage, spastic CP participants showed a narrow ${\dot{V}}_{E}$ range (71.4, 80.4, 88.3 L min^−^^1^) relative to CON (90.3, 95.7, 138.3 L min^−^^1^). Ataxic CP participants showed wide inter‐individual variability at this stage (48.3, 54.7, 112.9 L min^−^^1^), consistent with the functional heterogeneity of this subgroup (GMFCS I, I, III). One spastic CP participant showed a reduction in ${\dot{V}}_{E}$ from 26.0 to 25.1 L min^−^^1^ between the 120 and 140 bpm stages despite increasing exercise demand, accompanied by stable RF (23.8 vs. 23.4 breaths min^−^^1^) (Figure 2). Effect sizes were large across comparisons (|*r*| = 0.89–1.00); given *n* = 3 per group, complete rank separation is a mathematical near‐inevitability, and these values should not be interpreted as evidence of robust effect magnitude.

## 4 DISCUSSION

This exploratory study described intensity‐dependent ventilatory and cardiopulmonary response patterns across HR‐matched exercise stages in trained individuals with CP. Peak ${\dot{V}}_{O_{2}}$ appeared lower in CP participants than CON. Although prescribing exercise based on workload may attenuate the differences in oxygen consumption between individuals with and without CP, individuals with CP appeared to exhibit higher oxygen consumption even at rest (Appendix Table A1). Therefore, the reduced mechanical efficiency and increased energy cost associated with CP may be expected to persist during workload‐based exercise. In addition, prior literature suggests that response patterns at higher workload‐exercise stages may show greater divergence across individuals with CP groups than at lower stages (Balemans et al., 2013; Lundberg, 1978; Unnithan et al., 1996, 1998).

A possible ${\dot{V}}_{O_{2}}$ plateau relative to cardiac chronotropic demand was observed in spastic CP; whether this represents a peripheral metabolic, mechanical or central haemodynamic constraint cannot be determined without concurrent cardiac output measurement (Unnithan et al., 1996). Responses may differ when stages are matched by external workload, given the markedly higher metabolic cost of exercise at equivalent workloads in individuals with CP (Nardon et al., 2021; Unnithan et al., 1996).

*V*_T_ appeared to be reduced in participants with CP from rest across all exercise stages, consistent with previously reported reductions in *V*_T_ and chest wall excursion in this population (Balemans et al., 2013; Kwon & Lee, 2013; Leopando et al., 1999). At lower intensities, ${\dot{V}}_{E}$ appeared to be broadly preserved through a higher RF rather than an increase in *V*_T_, suggesting a compensatory breathing strategy rather than a primary ventilatory limitation. Regarding inter‐individual variability, participants with spastic CP showed a narrower ${\dot{V}}_{E}$ range at 180 bpm (71.4–88.3 L min^−^^1^) than CON participants (90.3–138.3 L min^−^^1^).

One spastic CP participant showed a reduction in ${\dot{V}}_{E}$ from 26.0 to 25.1 L min^−^^1^ between the 120 and 140 bpm stages, accompanied by a reduction in *V*_T_ (1.14–1.06 L) and stable RF (23.8 vs. 23.4 breaths min^−^^1^). The VO_2_ Master Pro has been reported to have a mean absolute percentage error of 5–12% for ${\dot{V}}_{E}$ across a range of ventilatory rates (Montoye et al., 2020); as the observed change of 0.9 L min^−^^1^ (3.5%) falls within this error range, it cannot be confidently interpreted as a true physiological change and is retained for descriptive completeness only. Additionally, the observed decline in O_2_ pulse at the final stage may reflect measurement error and/or insufficient control of workload across the stage, rather than a true physiological change.

Several limitations should be acknowledged. The small sample size (*n* = 3 per group) renders all patterns hypothesis‐generating only. The portable analyser did not provide respiratory exchange ratio (RER) or end‐tidal CO_2_, and direct haemodynamic measurements were not obtained; mechanistic interpretation of ventilatory efficiency and oxygen delivery is therefore precluded. Peak ${\dot{V}}_{O_{2}}$ was not obtained under standard maximal exercise test conditions (no RER criterion, ${\dot{V}}_{O_{2}}$ plateau verification or volitional exhaustion confirmation); it represents the highest value recorded at the final stage and should not be equated with ${\dot{V}}_{O_{2}\max}$ as reported in the comparative literature. In addition, HR was not consistently maintained within ±5 bpm of the target throughout each 2‐min stage. Therefore, the stage‐specific findings are presented descriptively and should not be interpreted as responses obtained under strictly matched HR conditions.

Sex was not matched between groups; all spastic CP participants were male, whereas controls and ataxic CP participants each included two females. There are established sex differences in peak aerobic capacity, with females averaging approximately 10–15% lower than males of comparable training status (Sparling, 1980). The observation that spastic CP participants nonetheless showed lower peak ${\dot{V}}_{O_{2}}$ than controls suggests that the CP‐related constraint on aerobic capacity may be larger than the sex‐related one. Recruitment was restricted to trained athletes, limiting generalizability to other functional levels and untrained individuals with CP.

In summary, trained individuals with CP demonstrated lower peak aerobic capacity than CON, with intensity‐dependent differences in O_2_ pulse trajectory, *V*_T_ and ventilatory pattern at higher exercise stages. These exploratory observations suggest that different patterning in ventilation and O_2_ pulse may capture one aspect of exercise‐related physiological stress in individuals with CP. These findings should be interpreted cautiously because of small sample size and further studies incorporating direct haemodynamic measurements, breath‐to‐breath ventilatory analysis and workload‐matched designs are needed.

## AUTHOR CONTRIBUTIONS

Conceptualization and funding acquisition: Min‐Goo Lee. Methodology: Min‐Goo Lee and Se‐Hee Park. Data curation, formal analysis, visualization, and writing—original draft: Se‐Hee Park. Investigation: Se‐Hee Park and Hwan Kim. Writing—review and editing: Se‐Hee Park, Hwan Kim, and Min‐Goo Lee. All authors were responsible for drafting the manuscript. All authors have read and approved the final version of this manuscript and agree to be accountable for all aspects of the work in ensuring that questions related to the accuracy or integrity of any part of the work are appropriately investigated and resolved. All persons designated as authors qualify for authorship, and all those who qualify for authorship are listed.

## CONFLICT OF INTEREST

The authors declare no conflicts of interest.

## GENERATIVE AI STATEMENT

No generative AI tools were used in the preparation of this manuscript.

## Data Availability

The data presented in this study are available on request from the corresponding author. The data are not publicly available due to privacy restrictions.

## 1

**TABLE A1:** Individual participant data across heart rate‐matched exercise stages.

Participant	Stage (bpm)	HR (bpm)	${\dot{V}}_{O_{2}}$ (mL kg^−^^1^ min^−^^1^)	${\dot{V}}_{E}$ (L min^−^^1^)	*V*_T_ (L)	RF (breaths min^−^^1^)	O_2_ pulse (mL beat^−^^1^)
CON‐1	Rest	74.0	2.9	5.6	1.17	4.9	3.38
CON‐1	120	122.3	16.9	36.9	1.94	20.0	11.89
CON‐1	140	144.5	26.1	48.5	2.44	20.5	15.55
CON‐1	160	163.8	32.8	63.5	2.42	26.6	17.23
CON‐1	180	186.7	49.3	138.3	2.43	57.5	22.69
CON‐2	Rest	75.1	4.4	10.4	0.81	13.3	3.70
CON‐2	120	123.7	12.3	24.1	0.81	30.6	6.10
CON‐2	140	142.7	18.6	31.7	1.35	25.1	7.94
CON‐2	160	161.4	25.6	47.3	1.31	37.2	9.67
CON‐2	180	189.4	39.8	90.3	1.81	50.3	11.99
CON‐3	Rest	71.8	3.1	5.3	0.44	11.9	2.45
CON‐3	120	118.8	19.4	32.0	1.26	26.7	10.27
CON‐3	140	141.3	27.0	43.0	1.57	28.3	12.05
CON‐3	160	167.6	34.6	57.0	1.76	33.1	13.02
CON‐3	180	188.2	48.5	95.7	1.88	51.0	16.23
SCP‐1	Rest	76.9	3.3	5.3	0.42	13.6	2.51
SCP‐1	120	114.5	18.5	28.1	0.93	31.0	9.52
SCP‐1	140	136.9	28.9	45.8	1.19	38.6	12.44
SCP‐1	160	160.5	35.8	65.8	1.22	54.0	13.14
SCP‐1	180	165.8	33.9	71.4	1.29	55.8	12.07
SCP‐2	Rest	93.3	3.8	6.2	0.37	16.8	2.13
SCP‐2	120	122.9	16.6	26.0	1.14	23.4	7.00
SCP‐2	140	147.0	25.9	25.1	1.06	23.8	9.13
SCP‐2	160	168.3	34.1	43.4	1.37	32.5	10.52
SCP‐2	180	192.9	44.8	80.4	1.76	45.4	12.09
SCP‐3	Rest	70.4	3.7	8.8	0.41	21.3	4.04
SCP‐3	120	128.6	16.6	39.3	0.93	43.5	9.89
SCP‐3	140	141.6	20.5	50.0	1.07	47.2	11.04
SCP‐3	160	167.7	26.3	67.3	1.20	58.0	12.00
SCP‐3	180	178.3	31.7	88.3	1.19	74.5	13.61
ACP‐1	Rest	88.8	3.6	7.7	0.45	17.4	2.77
ACP‐1	120	112.8	10.8	21.4	0.81	26.5	6.49
ACP‐1	140	127.0	13.6	29.5	0.88	33.6	7.29
ACP‐1	160	166.3	30.6	62.7	1.50	42.3	12.51
ACP‐1	180	188.8	35.3	112.9	1.68	67.2	12.71
ACP‐2	Rest	72.1	3.6	6.3	0.33	18.7	2.64
ACP‐2	120	121.2	11.5	13.8	0.69	20.3	5.03
ACP‐2	140	147.1	16.7	22.3	0.71	31.1	6.02
ACP‐2	160	163.1	20.3	29.0	0.93	31.4	6.61
ACP‐2	180	190.3	32.3	48.3	1.17	40.9	9.01
ACP‐3	Rest	81.5	2.3	8.4	0.27	30.8	1.76
ACP‐3	120	123.5	12.2	29.2	0.78	37.4	6.20
ACP‐3	140	142.9	19.1	40.3	0.87	46.1	8.40
ACP‐3	160	157.2	23.1	47.4	0.96	49.4	9.23
ACP‐3	180	165.7	28.3	54.7	0.93	59.5	10.75

*Note*: Stage denotes target heart rate (bpm); achieved HR is presented in the HR column. Values are rounded to one decimal place.

Abbreviations: ACP, ataxic cerebral palsy; CON, non‐disabled control; HR, heart rate; RF, respiratory frequency; SCP, spastic cerebral palsy; ${\dot{V}}_{E}$, minute ventilation; ${\dot{V}}_{O_{2}}$, oxygen uptake; *V*_T_, tidal volume.
